# Effect of Exogenous γ-Aminobutyric Acid (GABA) on the Growth, Photosynthetic Pigment, Antioxidant and GABA Metabolism of *Festuca arundinacea* (Tall Fescues) Under Cadmium Stress

**DOI:** 10.3390/plants14030383

**Published:** 2025-01-27

**Authors:** Wan Geng, Yangyang Zhang, Caihua Li, Guilong Song, Shengqing Shi

**Affiliations:** 1 College of Grassland Science, Beijing Forestry University, Beijing 100083, China; wanana771@163.com; 2 Beijing Geological and Mineral Exploration and Development Group Co., Ltd., Beijing 100016, China; 3 Shijiazhuang Academy of Agriculture and Forestry Science, Shijiazhuang 050040, China; 4 State Key Laboratory of Tree Genetics and Breeding, Key Laboratory of Tree Breeding and Cultivation of the State Forestry Administration, Research Institute of Forestry Research, Chinese Academy of Forestry, Beijing 100091, China

**Keywords:** γ-aminobutyric acid, cadmium stress, antioxidants, GABA shunt, plant growth

## Abstract

γ-Aminobutyric acid (GABA), an endogenous amino acid widely found in living organisms, has important functions in plants such as regulating growth and development, maintaining carbon and nitrogen nutrient balance, and coping with adversity. In this study, we investigated the effects of exogenous 0.5 mmol/L GABA on the growth, antioxidant metabolism, and GABA shunt metabolism of tall fescue under 20 μmol/L Cd stress, using tall fescue (*Festuca arundinacea*) ‘Ruby II’ under hydroponics conditions. The results showed that (1) applying GABA for 3, 7, 11, and 15 d under Cd stress inhibited Cd transport from roots to leaves and promoted plant height, alleviating the effects of Cd stress on plant growth. (2) Exogenous 0.5 mmol/L GABA had an interesting regulatory effect on the activation of the antioxidant enzyme system induced by stress at different stages, which was accompanied by a decrease in malondialdehyde (MDA) contents and alleviated the degree of cell membrane lipid peroxidation under cadmium stress. Specifically, peroxidase (POD) enzyme activity reactions initially responded on the 3rd and 7th days of stress, and the changes in catalase (CAT) enzyme activities concentrated on the 11th and 15th days of the later stage. Ascorbate peroxidase (APX) enzyme was active throughout the whole stress period in the roots. Multiple factorial analyses further proved that the antioxidant pathway strongly influenced the survival and growth of tall fescue under stress in the presence of GABA. (3) Application of exogenous GABA activated the branching pathway for GABA synthesis from Glu decarboxylation (GABA shunt) with a higher contribution in the leaves, which induced changes in glutamate content, and plants maintained a higher endogenous GABA content and signal to regulate the plant antioxidant system and reduce cell membrane damage, thus improving the tolerance of plants to Cd stress.

## 1. Background

Heavy metal-contaminated soils pose a serious threat to food safety and are a potential global agricultural and environmental problem [1]. Cadmium (Cd) is currently the most serious heavy metal contaminating surface soils, which enters the plant body and usually changes its cell membrane permeability, inhibits photosynthesis, and induces oxidative stress, causing peroxidation of plant cell membrane lipids [2,3]. Plant photosynthesis is sensitive to heavy metals, and the transfer of Cd to leaves can disrupt photosynthetic reaction centers, degrade photosynthetic pigments and reduce photosynthetic efficiency, thus affecting leaf photosynthesis and reducing plant biomass [4,5,6]. The chlorophyll content is an important indicator of plant photosynthesis, which plays a role in photosynthesis by absorbing, transferring, and converting light energy, and can to a certain extent reflect the level of photosynthesis in plants. Studies have shown that higher concentrations of Cd contamination in the soil can significantly reduce the chlorophyll a, chlorophyll b, and carotenoid contents of *Salix variegata* Franch [7] and maize (*Zea mays*) [8]. In addition, the accumulation of excessive reactive oxygen species (ROS) can aggravate the degree of cell membrane lipid peroxidation and even lead to cell death. At low levels, ROS are signaling molecules, and in excess, ROS can cause damage to biomolecules such as lipids, proteins, and DNA, disrupting cell integrity and ultimately leading to cell death [9]. In recent years, studies have demonstrated that γ-aminobutyric acid (GABA) can effectively mitigate the toxic effects of salinity, drought, and heavy metal stresses on plants, mainly including the effects of GABA on germination, photosynthetic performance, and oxidative damage in stressful environments [10,11,12].

GABA, a naturally occurring nonprotein amino acid, is widely distributed in the biological community [13,14]. Glutamate (Glu) decarboxylation is one of the pathways for the synthesis and conversion of GABA, and synthesized GABA enters the tricarboxylic acid cycle after being catalyzed by γ-aminobutyric acid transferase (GABA-T) and succinate semialdehyde dehydrogenase (SDH), a process known as GABA-branched metabolism [15,16]. GABA is an important component of the free amino acids and is present in low concentrations in plant tissues, and its levels are altered in response to many endogenous factors [17]. As GABA has been investigated, an increasing number of its functions have been explored. As a signaling molecule, GABA was shown to be necessary and sufficient in guarding cells for reducing stomatal opening and transpiration water loss, which improves water use efficiency and stress tolerance [18]. The anion flux of the malic acid transporter (*ALMT*) activated by plant aluminum is activated by anions and negatively regulated by GABA [17]. Results showed that the germination rate, vigor index, fresh weight, dry weight, and radicle lengths of tomato (*Solanum lycopersicum* L.) seeds pretreated with GABA (10–200 μM) significantly increased under Cd stress [19]. Glutamate decarboxylase (GAD) is a key enzyme in GABA synthesis. Apple (*Malus hupehensis*) roots overexpressing *MdGAD1* accumulated less Cd in the root system compared to the control, and 0.5 mM exogenous GABA significantly reduced the expression of Cd uptake under Cd stress, effectively reducing the toxicity of Cd [20].

GABA changes in plants are mainly resistant to environmental stresses, and GABA may play a dual role as a metabolite and signaling molecule under different abiotic adversity stresses [21]. The exogenous application of GABA can enhance plant tolerance to heavy metals, just as the exogenous application of GABA plays a role in enhancing tolerance to aluminum stress in *Hybrid Liriodendron* (L. *chinense × tulipifera*) by promoting organic acid transport and maintaining cellular redox and osmotic balance [22]. Yang et al. found that Cd (50 mg kg^−1^) treatment increased the SOD, POD, and CAT activities and the MDA content in Dahurian wildrye grass (*Elymus dahuricus*) [23]. Growth inhibition, photosynthetic damage, accumulation of excess ROS, and cell membrane damage caused by Cd stress reduced Cd stress tolerance in *Platycladus orientalis* seedlings [24]. Khanna’s study on wheat found that exogenous GABA reversed the adverse effects of salt stress by enhancing the antioxidant system, N-S assimilation, and other metabolic processes, while GABA regulated NO production to promote photosynthesis and growth [25]. Many experiments have demonstrated the positive effects of exogenous GABA on the antioxidants, photosynthesis, and growth of plants under adverse stress, and additional experiments are needed to help us further understand the response and utilization of GABA in plants.

Tall fescue (*Festuca arundinacea*) is a common cool-season turfgrass with high ornamental value and strong stress resistance, and is a common plant material in urban greening. A study has shown that tall fescue has good Cd enrichment capacity and strong Cd tolerance, making it an ideal phytoremediation material for the ecological remediation of heavy metal-contaminated soil [26]. GABA as a safe, easy-to-obtain, and efficient exogenous regulator has not been applied to heavy metal toxicity ecological restoration. This study clarifies the response mechanism of tall fescue with externally applied GABA to cadmium stress, which can provide a theoretical basis for the application of the ecological remediation of tall fescue to extract heavy metals.

## 2. Results

### 2.1. Effects of Exogenous GABA on the Growth of Tall Fescue Under Cadmium Stress at Different Concentrations

**Figure 1:**
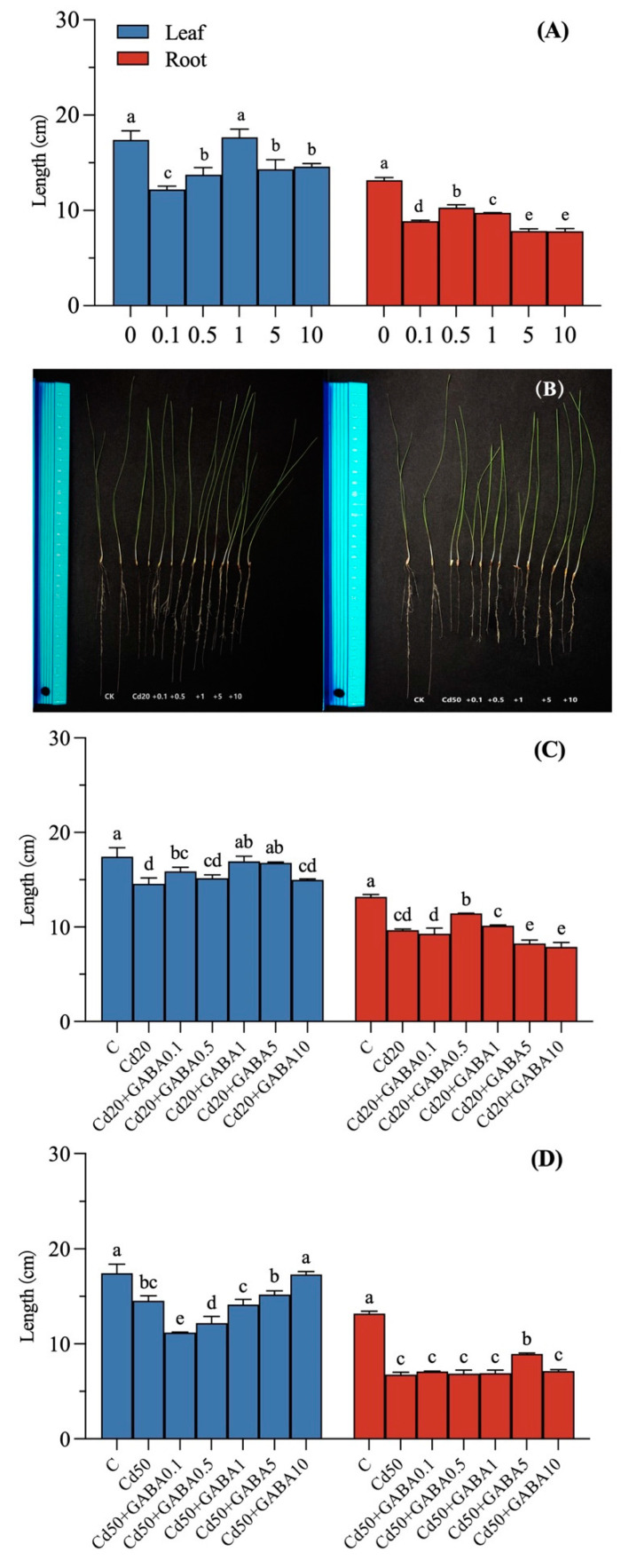
Effects of exogenous GABA on the growth of leaves and roots in tall fescue under Cd stress. (**A**) Length changes in response to the different concentrations of GABA; (**B**) changes in the different concentrations of GABA phenotypes under Cd stress, from left to right, two plants each of C, Cd20, Cd20 + 0.1, 0.5, 1, 5, or 10 mmol/L GABA; and Cd50, Cd50 + 0.1, 0.5, 1, 5, or 10 mmol/L GABA; (**C**) length changes in response to the different concentrations of GABA under 20 μmol/L Cd stress; (**D**) length changes in the different concentrations of GABA under 50 μmol/L Cd stress at 11 days of normal and Cd stress conditions of tall fescue. Vertical bars above columns represent standard errors. The different letters above the columns indicate a significant difference (n = 3 and *p* < 0.05).

The effects of different concentrations of exogenous GABA on the leaf and root lengths of tall fescue are shown in Figure 1A. Compared with C, the leaf length was least inhibited under 1 mmol/L GABA treatment, while the root length was least inhibited under the 0.5 mmol/L GABA treatment, with only a 21.90% reduction in root length. The inhibitory effect on the plants increased and then decreased with increasing external GABA concentration. The phenotypic effects of the different concentrations of exogenous GABA treatments on tall fescue leaf and root length under different Cd concentration stresses are shown in Figure 1B. Compared with C, the leaf and root lengths under 20 μmol/L Cd^2+^ treatment decreased by 16.46% and 26.68%, respectively, reaching significant differences (Figure 1C). Compared to other concentrations of GABA, external application of 1 mmol/L GABA had the best promotion effect on plant height under 20 μmol/L Cd^2+^ stress, with a significant increase of 16.23% compared to the Cd 20 treatment. The underground length changes were different; the difference was most significant under the 0.5 mmol/L GABA treatment, and the root length increased by 18.31%. Under 50 μmol/L Cd^2+^ stress, the belowground parts of plants were significantly inhibited as shown in Figure 1D, and the inhibition was not alleviated by different concentrations of exogenous GABA, except for 5 mmol/L GABA. Combining the results of the different exogenous GABA and Cd applications resulted in a group of 0.5 mmol/L GABA and 20 μmol/L Cd for subsequent experiments.

### 2.2. Effects of Exogenous GABA on Cadmium Content of Tall Fescue Under Cadmium Stress

**Figure 2:**
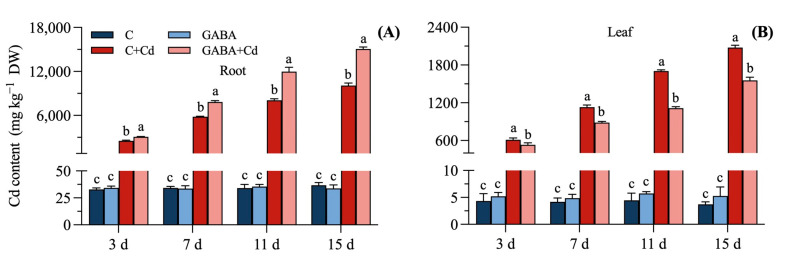
Effects of exogenous GABA on Cd content of tall fescue under cadmium stress. Cd content changes in the (**A**) roots and (**B**) leaves, under 20 μmol/L cadmium stress at 15 days in tall fescue. Vertical bars above columns represent standard errors. The different letters above the columns indicate a significant difference (n = 3 and *p* < 0.05). C, control; GABA, control treated with GABA (0.5 mmol/L); C + Cd, Cd stress; GABA + Cd, cadmium-stressed plants treated with GABA.

Treatment with Cd increased the amount of Cd in tall fescue (Figure 2A,B). After 15 days of Cd stress, the Cd content in the Cd + Cd treatment root was 274.57 times higher than that in the C treatment, and the Cd content in the leaves was 558.31 times higher than that in the C treatment. However, the GABA treatment did not significantly affect Cd content compared to C. Under Cd stress treatments, the root Cd content increased by 21.64% at 3 d, 34.45% at 7 d, 48.43% at 11 d, and 49.61% at 15 d in GABA + Cd treatment compared to C + Cd treatment as treatment time increased. In contrast, the amount of Cd in the leaves was significantly reduced by 13.25% at 3 d, 21.77% at 7 d, 34.67% at 11 d, and 25.10% at 15 d in the GABA + Cd treatment compared to the C + Cd treatment.

### 2.3. Effects of Exogenous GABA on Photosynthetic Pigments on Tall Fescue Under Cadmium Stress

**Figure 3:**
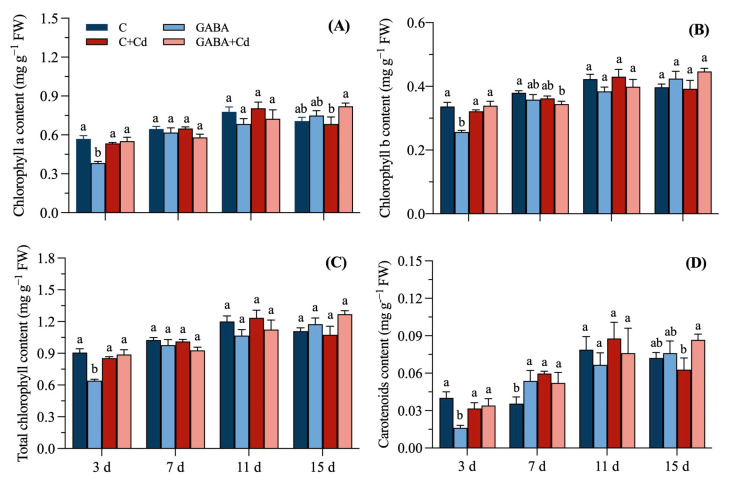
Effects of exogenous GABA on photosynthetic pigments of tall fescue under cadmium stress. (**A**) Chlorophyll a content, (**B**) chlorophyll b content, (**C**) total chlorophyll content, and (**D**) carotenoid content under 20 μmol/L cadmium stress at 15 days in tall fescue. Vertical bars above columns represent standard errors. The different letters above the columns indicate a significant difference (n = 3 and *p* < 0.05). C, control; GABA, control treated with GABA (0.5 mmol/L); C + Cd, Cd stress; GABA + Cd, cadmium-stressed plants treated with GABA.

Under different treatment conditions, the contents of chlorophyll a, chlorophyll b, total chlorophyll, and carotenoids in the leaves of tall fescue are shown in Figure 3. Under the condition of GABA (0.5 mmol/L) treatment, the chlorophyll a, chlorophyll b, total chlorophyll, and carotenoid contents of tall fescue leaves were significantly reduced by 32.75%, 23.83%, 29.22%, and 59.58%, respectively, compared with the C treatment on the 3rd day. While plants receiving the GABA + Cd treatment had substantially more photosynthetic pigment than plants receiving the GABA treatment, the amounts of chlorophyll a, chlorophyll b, total chlorophyll, and carotenoids increased by 44.19%, 32.12%, 38.59%, and 109.82%, respectively. Compared with the C + Cd treatment, in GABA + Cd treated leaves, only the chlorophyll a and carotenoid contents decreased significantly on the 15th day of treatment.

### 2.4. Effects of Exogenous GABA on the Cell Membrane Damage and Antioxidant Activity of Tall Fescue Under Cadmium Stress

**Figure 4:**
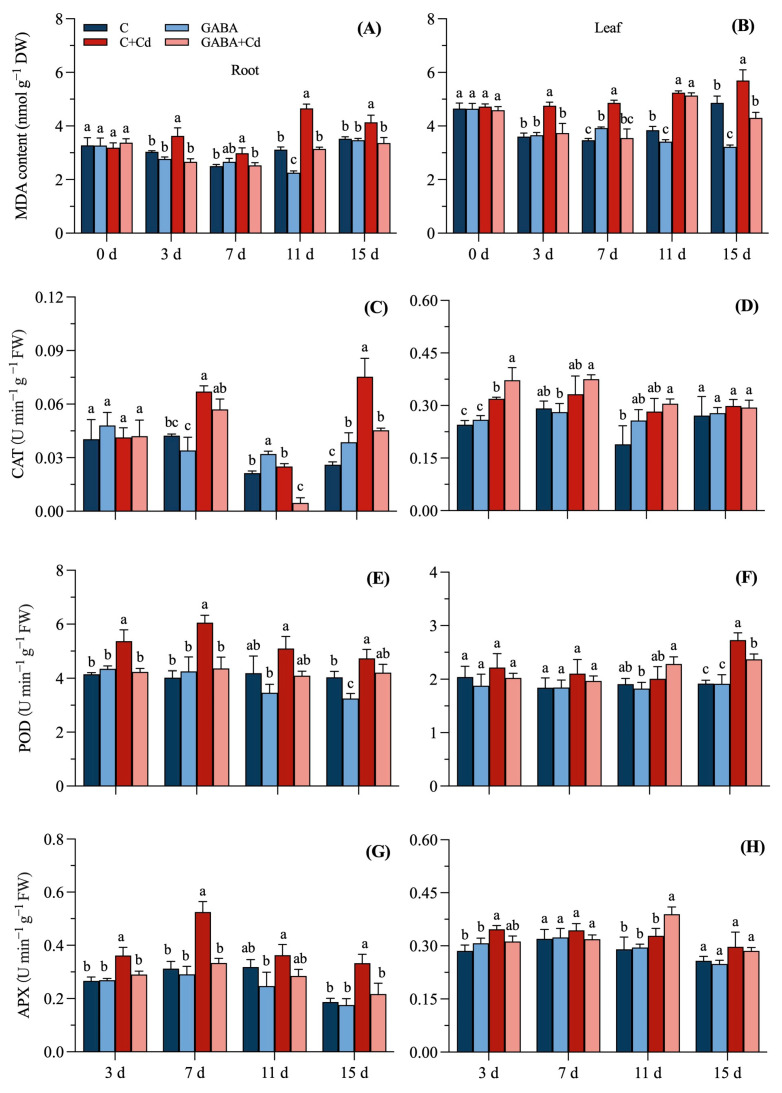
Effects of exogenous GABA on the cell membrane damage and antioxidant of tall fescue under cadmium stress. MDA content change in the (**A**) roots and (**B**) leaves, catalase (CAT) activity in the (**C**) roots and (**D**) leaves, peroxidase (POD) activity in the (**E**) roots and (**F**) leaves, and ascorbate peroxidase (APX) activity in the (**G**) roots and (**H**) leaves under 20 μmol/L cadmium stress at 15 days in tall fescue. Vertical bars above columns represent standard errors. The different letters above the columns indicate a significant difference (n = 3 and *p* < 0.05). C, control; GABA, control treated with GABA (0.5 mmol/L); C + Cd, Cd stress; GABA + Cd, cadmium-stressed plants treated with GABA.

Under GABA treatment, the MDA content in roots increased by 6.45% at 7 d, decreased by 27.59% at 11 d, and was not significantly different at 15 d compared to the C treatment (Figure 4A). The MDA content in GABA-treated leaves of tall fescue showed an increase of 13.18% on day 7 and significant decreases of 11.19% and 33.70% on days 11 and 15 compared to the C treatment (Figure 4B). Under the C + Cd treatment, the MDA content of both the leaves and roots of tall fescue increased significantly compared to the C treatment, by 32.09% at 3 d, 40.31% at 7 d, 36.36% at 11 d, and 17.13% at 15 d in the leaf portion and 19.47% at 3 d, 19.36% at 7 d, 49.31% at 11 d, and 17.56% at 15 d in the roots, respectively. Compared to that in the C + Cd group, the MDA content of both the leaves and roots of tall fescue was significantly reduced in the GABA + Cd group by 21.47%, 27.07%, 2.05%, and 24.53% in leaves and 26.67%, 15.32%, 32.45%, and 18.83% in roots, respectively. Compared to that in the C group, the leaf MDA content of tall fescue in the GABA + Cd group only increased significantly on the 11th day of treatment.

The CAT activity of tall fescue roots was significantly affected by Cd stress on days 7 and 15, with the greatest change occurring on day 15 with a significant increase of 189.74%. The root CAT activity under GABA + Cd treatment significantly decreased by 81.33% and 39.82% on days 11 and 15, respectively, compared to that under C + Cd treatment (Figure 4C,D). CAT activity in leaves treated with GABA increased significantly by 76.43% compared to that in leaves of the C treatment only on day 11 of the treatment; roots showed significant changes of 50.00% and 48.72% on days 11 and 15, respectively. CAT activity in leaves of tall fescue was significantly increased by 16.61% at 3 d.

The POD activities in the roots and leaves of tall fescue under different treatment conditions are shown in Figure 4E,F. Cd stress enhanced POD activity in tall fescue roots, reaching significant differences at 3 d and increasing by 29.41%, 50.58%, 21.64%, and 17.18% at 3 d, 7 d, 11 d, and 15 d, respectively; leaf POD activity was significantly higher than that in the C treatment only on day 15. Compared to the C + Cd treatment, the POD activity of tall fescue leaves under the GABA + Cd treatment was significantly lower at 15 d, whereas the POD activity of roots was significantly different at 3 d of treatment, decreasing by 21.12%, 28.09%, 19.65%, and 11.00% at 3 d, 7 d, 11 d, and 15 d, respectively. The effect of GABA treatment on POD activity in plant roots varied with treatment time, and POD activity was significantly reduced by 21.17% and 24.11% at 11 d and 15 d of treatment, respectively.

The application of GABA had no significant effect on the root and leaf APX activities of tall fescue, but C + Cd stress promoted the enhancement of APX activity in tall fescue, which was significantly increased by 21.35% in leaves after only 3 days of stress and by 67.98%, 13.93%, and 77.58% in roots at 7 d, 11 d, and 15 d of stress (Figure 4G,H). The APX activity of roots was significantly reduced in the GABA + Cd treatment by 36.34%, 21.32%, and 34.87% at 7 d, 11 d, and 15 d of treatment, respectively; the APX activity of tall fescue leaves under GABA + Cd treatment was significantly higher at 11 d of treatment, 18.49% higher than that under the C + Cd treatment.

### 2.5. Effects of Exogenous GABA on the Major Metabolites of the GABA Shunt, and Related Enzyme Activities of Tall Fescue Under Cadmium Stress

**Figure 5:**
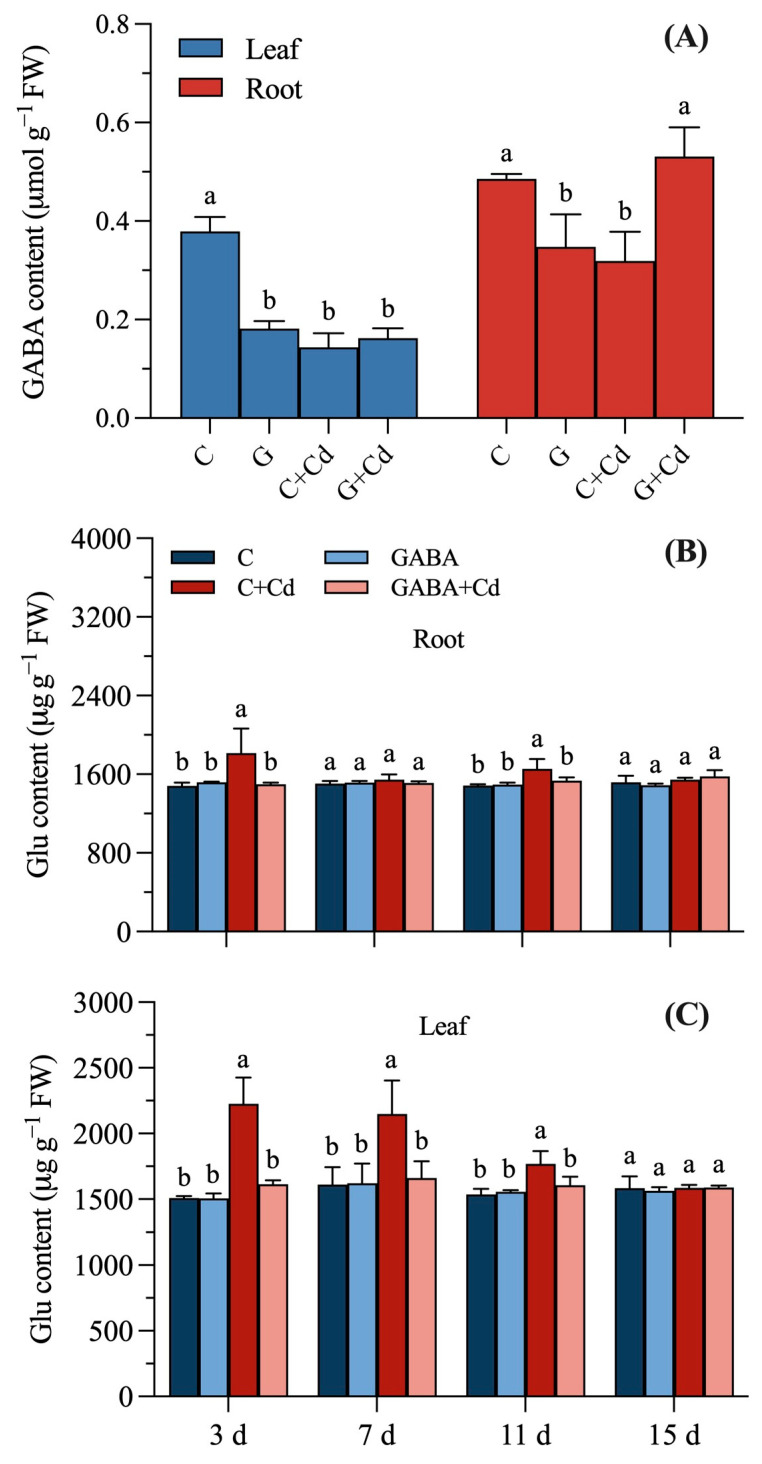
Effects of exogenous GABA on the major metabolites of the GABA shunt of tall fescue under cadmium stress. (**A**) GABA content at 11 days, (**B**) glutamate (Glu) content in the roots, and (**C**) Glu content in the leaves of tall fescue under 20 μmol/L cadmium stress. Vertical bars above columns represent standard errors. The different letters above the columns indicate a significant difference (n = 3 and *p* < 0.05). C, control; GABA, control treated with GABA (0.5 mmol/L); C + Cd, Cd stress; GABA + Cd, cadmium-stressed plants treated with GABA.

The GABA content in tall fescue roots and leaves at 11 d was significantly reduced by 51.98% and 28.37%, respectively, after GABA application compared to the C treatment (Figure 5A). There was no significant difference in the GABA content of leaves under the GABA + Cd treatment compared with the C + Cd treatment, while the GABA content of roots increased significantly by 66.44%.

GABA treatment had no significant effect on the accumulation of Glu in the roots and leaves of tall fescue (Figure 5B,C). The C + Cd treatment promoted the accumulation of Glu in the roots and leaves of tall fescue, with a significant increase of 22.56% and 11.59% in the roots at 3 d and 11 d, and a significant increase of 47.54%, 33.25%, and 15.20% in the leaves at 3 d, 7 d, and 11 d, respectively. Compared with the C + Cd treatment, the Glu content of the leaves of tall fescue under the GABA + Cd treatment showed a decreasing trend. The Glu content in the roots decreased significantly on days 3 and 11, by 17.42% and 7.40%, respectively, while that in the leaves decreased significantly on days 3, 7, and 11, by 27.48%, 22.73%, and 9.27%, respectively. There was no significant difference in Glu content in the leaves and roots of tall fescue under the GABA + Cd treatment compared to the C treatment.

**Figure 6:**
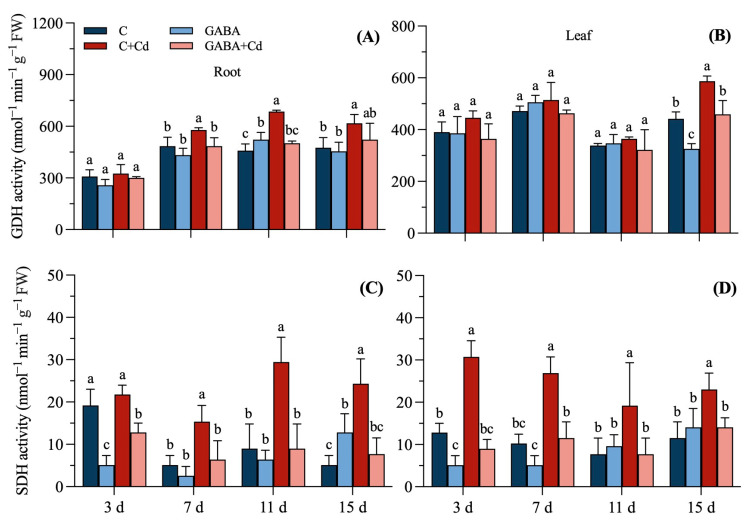
Effects of exogenous GABA on related enzyme activities of tall fescue under cadmium stress. (**A**) Glutamate dehydrogenase (GDH) activity in the roots, (**B**) GDH activity in the leaves, (**C**) succinate dehydrogenase (SDH) activity in the roots, and (**D**) SDH activity in the leaves of tall fescue under 20 μmol/L cadmium stress. Vertical bars above columns represent standard errors. The different letters above the columns indicate a significant difference (n = 3 and *p* < 0.05). C, control; GABA, control treated with GABA (0.5 mmol/L); C + Cd, cadmium stress; GABA + Cd, cadmium-stressed plants treated with GABA.

There was a significant increase of 14.02% at 11 d of treatment (Figure 6A). Leaf GDH activity was not significantly different at 3 d, 7 d, and 11 d and significantly decreased by 26.21% at 15 d. Cd stress forced an increase in GDH activity in the roots and leaves of tall fescue (Figure 6A,B), and the roots’ GDH activity increased significantly on 7th, 11th, and 15th days, increasing by 19.47%, 49.53%, and 29.73%, respectively. The leaves’ GDH activity significantly increased by 33.01% at 15 d. The GDH activities of the roots and leaves of fescue under the GABA + Cd treatment did not differ significantly from those under the C treatment. The GDH activity of roots and leaves under the GABA + Cd treatment was low, and the root GDH activity was significantly reduced by 16.30%, 26.88%, and 15.28% compared to the C + Cd treatment on days 7, 11, and 15, respectively. The leaves’ GDH activity significantly reduced by 21.90% on day 15 of treatment.

Under different treatment conditions, the SDH activity of the roots of tall fescue is shown in Figure 6C. The SDH activity of plant roots under GABA treatment was significantly reduced by 73.00% on day 3 and increased significantly by 1.5 times on day 15 compared with the C treatment. The SDH activity of roots under cadmium stress increased significantly at 7 d, 11 d, and 15 d of treatment, and the largest increase was at 15 d, which was 3.75 times that of the C treatment. Under the GABA + Cd treatment, the SDH activities of roots at 3 d, 7 d, 11 d, and 15 d were reduced by 41.18%, 58.33%, 69.57%, and 68.42%, respectively, compared with the C + Cd treatment. The SDH activity of the leaves decreased significantly in GABA treatments at 3 d and 7 d, which were reduced by 60.00% and 50.00%, respectively (Figure 6D). The C + Cd treatment significantly increased the leaves’ SDH activity at 3 d, 7 d, 11 d, and 15 d of treatment by 140.00%, 162.50%, 150.00%, and 100.00%, respectively. Compared with the C + Cd treatment, the SDH activity was reduced by 70.83%, 57.14%, 60.00%, and 38.89% at 3 d, 7 d, 11 d, and 15 d of tall fescue leaves under the GABA + Cd treatment.

### 2.6. MFA of Tall Fescue to Exogenous GABA Supplied Under Cd Stress

**Figure 7:**
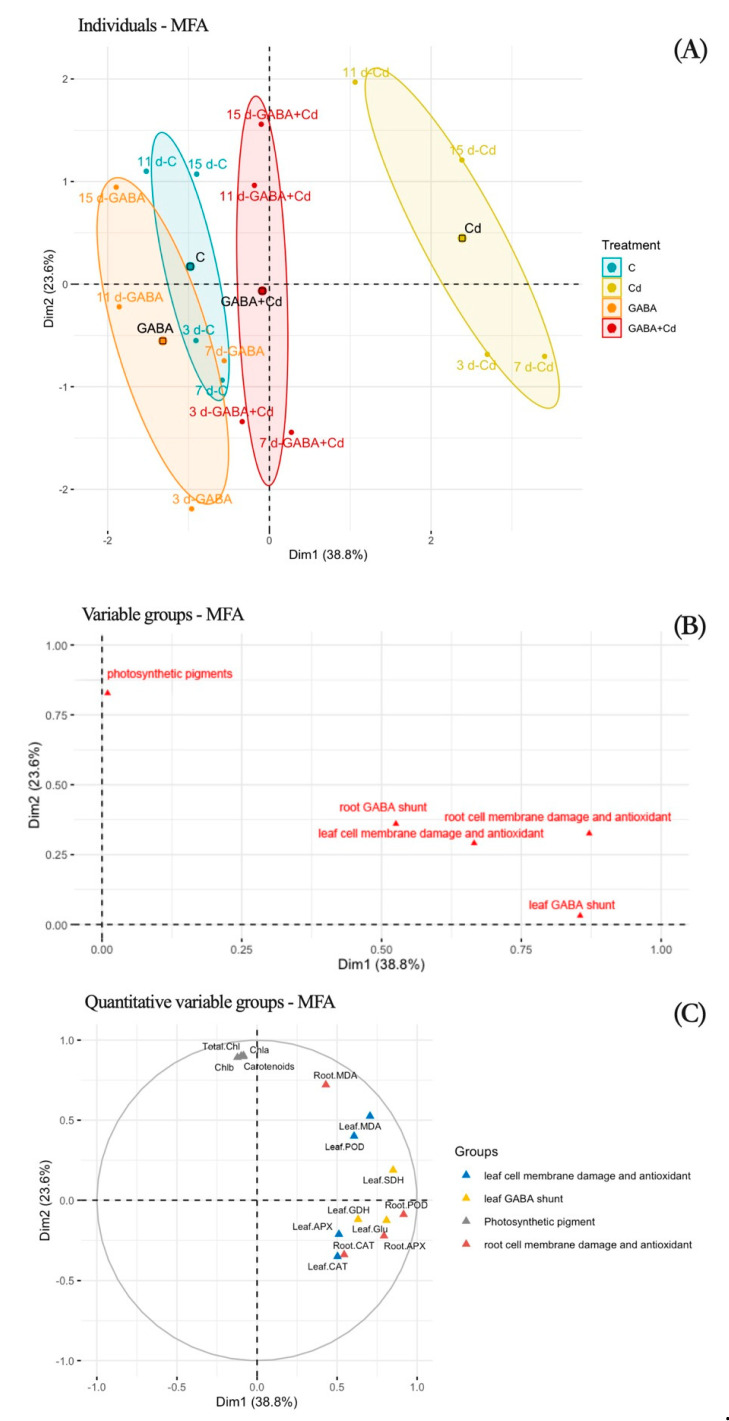
MFA of 18 parameters related to photosynthetic pigment, antioxidative system, and GABA metabolism in tall fescue under 20 μmol/L cadmium treatment with GABA applications. (**A**) The sample space of MFA shows the effects of exogenous GABA under Cd stress; (**B**) the functional group space of MFA shows the contribution of each functional group; (**C**) the parameter space of MFA shows the contribution of each parameter. C, control; GABA, control treated with GABA (0.5 mmol/L); C + Cd, Cd stress; GABA + Cd, cadmium-stressed plants treated with GABA.

The indices of root and leaf stress tolerance are affected differently by different treatments and times of the day. To account for the impacts of all the pathways in one study and to identify meaningful relationships, we used MFA on all measured photosynthetic pigments and antioxidant indicators with various pathways. The analysis revealed that the first two components, Dim1 and Dim2, explained 62.4% of the overall variation in these data (Figure 7). Dim 1 accounted for 38.8% of the variance and Dim 2 accounted for 23.6% of the variance. It was noted as a major difference between medication treatments as the research revealed that participants received various pharmacological treatments grouped together rather than receiving them at various periods. Additionally, because it was segregated into C, GABA, and GABA + Cd treatments in the MFA sorting, the Cd was the most typical in our experiment (Figure 7A). We found that most functional groups had large weights on Dim 1, suggesting that most of these parameters were significantly impacted by exogenous GABA after analyzing the group contribution space (Figure 7B). The MFA axis itself may be understood. The first one, which can be summed up as a positive contribution on the first axis and is related to GABA shunting and cell membrane damage, has the most positive correlations, including those of roots and leaves. However, Dim 2 was driven by photosynthetic pigments and had a larger weight (>0.75) than the other groups. A more thorough investigation revealed that plant MDA (both root and leaf) and photosynthetic pigments (total chlorophyll, chlorophyll a, chlorophyll b, and carotenoids) were the primary influences on Dim 2 variance (Figure 7C).

## 3. Discussion

As a nonessential heavy metal element of plants, Cd ions produce a series of poisons after being absorbed, thereby inhibiting plant growth. Plant roots are first affected by cadmium as the first site of contact with Cd [27]. Cadmium stress shortens the root system, and the effect of 50 μmol/L cadmium on tall fescue roots is greater than that of 20 μmol/L cadmium. The response of the aerial part to cadmium stress concentration is less sensitive than that of the root system, but cadmium stress also has an inhibitory effect on plant height. Li et al. found that exogenous 0.5 mmol/L GABA was effective in mitigating cadmium toxicity in apple seedlings compared to roots under Cd stress [20]. The balance of GABA is important for both plant growth and disease resistance, with plants being more susceptible to disease in the absence of GABA and excessive accumulation of GABA inhibiting normal plant growth [28]. Under normal circumstances, exogenous GABA has an inhibitory effect on the growth of tall fescue but has a relieving effect on growth inhibition caused by cadmium stress, and external 0.5 mmol/L GABA can alleviate the inhibitory effect of 20 μmol/L cadmium ions on the root system of tall fescue. The optimal GABA concentration is 0.5 mmol/L. In addition, the effect on the leaves varies with the concentration of GABA. The external application of low concentrations of GABA (0.1, 0.5 mmol/L) did not alleviate the inhibition of leaf length by 50 μmol/L cadmium stress. Our MFA further uncovered a unique GABA role related to tall fescue adaptations to cadmium conditions (Figure 7A).

Photosynthesis is the basic function of plants to maintain their normal growth and development; chlorophyll and carotenoids are the main photosynthetic pigments in plants, and their content levels can measure the strength of plant photosynthesis [29,30]. Chlorophyll a and chlorophyll b are the key constituent pigments of chlorophyll; the former is responsible for converting light energy into chemical energy by the light reaction center, the latter is responsible for capturing and transmitting light energy, and carotenoids are responsible for protecting chlorophyll, absorbing light energy, and quenching ROS [31,32]. The pre-application (3 days) of externally applied GABA may disrupt photosynthetic pigment synthesis by regulating stomatal opening [33], so chlorophyll a, chlorophyll b, total chlorophyll, and carotenoid contents were significantly lower in the GABA treatment than CK. The chlorophyll a, chlorophyll b, and carotenoid contents of tall fescue leaves treated with Cd stress were reduced at 15 d, and similar results were also confirmed in maize; soil Cd contamination significantly reduced chlorophyll a, chlorophyll b, and total chlorophyll contents in maize [34]. Cd stress can inhibit the activity of chlorophyll synthase enzymes and block the synthesis of photosynthetic pigments such as carotenoids and chlorophyll, while Cd poisoning of the plant causes an increase in oxygen radicals in the chloroplast, which may disrupt the composition of the chloroplast membrane and the structure of the photosynthetic pigment–protein complex, causing a reduction in the photosynthetic capacity of tall fescue. GABA-pretreated tall fescue exhibited significantly higher chlorophyll a and carotenoid contents than untreated plants in response to cadmium stress, indicating the positive effects of GABA in alleviating photoinhibition. We suggest that this may be an important role of carotenoids as precursors for plant stress resistance signal synthesis, such as abscisic acid (ABA) and strigolactone (SL), in defense against photosynthetic oxidative damage and plant tolerance. This provides evidence for the results of MFA for the photosynthetic pigment group (Figure 7B). In addition, GABA inhibiting the transport of cadmium from roots to leaves to ensure the basic function of leaves is also the reason why the chlorophyll a and carotenoid contents of GABA-pretreated tall fescue under Cd stress were significantly higher than Cd-treatment plants.

Excess Cd ions in the soil increase the production of ROS in plants, causing cellular lipid peroxidation reactions, and the accumulation of the lipid peroxidation product MDA causes oxidative damage [35]. Similarly to the results of Moussa and El-Gamal in wheat (*Triticum aestivum*) and Jia H et al. in Arabidopsis (*Arabidopsis thaliana*), the MDA content was significantly increased in tall fescue under Cd treatment alone, which could be a result of oxidative damage to cell membranes due to Cd toxicity [36,37]. The MDA content in both leaves and roots subjected to Cd stress after the addition of GABA was significantly lower than that under Cd treatment alone, confirming that GABA application could alleviate the cellular damage suffered by tall fescue under Cd stress. This result is inextricably linked to changes in antioxidant enzymes. The scavenging of free radicals can be achieved by the antioxidant enzymes SOD, POD, and CAT, and plants enhance their adaptation to Cd stress adversity by regulating the activity of antioxidant enzymes [24]. The APX, CAT, and POD enzyme activities of Cd-treated tall fescue showed an increasing trend, and the roots were more sensitive to Cd stress than the leaves. The APX and POD enzyme activities of G + Cd-treated tall fescue roots were significantly reduced compared to the Cd treatment, and the enhanced APX and POD enzyme activities of tall fescue roots caused by Cd stress were inhibited by the external application of GABA. It has been shown in several studies that GABA is involved in mediating the activity of antioxidant enzymes and activating the antioxidant defense system of plants under stresses such as salinity, heat, cold, and heavy metals [38,39,40,41]. Externally applied GABA reduced APX and POD enzyme activities in the roots of tall fescue under Cd stress, which might be related to the accumulation of more Cd content in roots. In this work, the response time of antioxidant enzymes to Cd stress was different in exogenous GABA-treated tall fescue roots. The CAT enzyme activity changes are concentrated in the later stages of stress, POD enzyme activity responds earliest, and APX enzyme activity is active throughout the whole stress period. This could be connected to how much stress plant tissues are subjected to and how long the reaction lasts. Our MFA identified antioxidant enzyme activity as an important contributor to plant responses to Cd stress (Figure 7C). In addition, under stress conditions, GABA is utilized more for providing energy for plant growth than for the production of antioxidant enzymes [42].

Glutamate (Glu) is an essential plant amino acid that acts as a N donor in biosynthesis and a synthetic GABA precursor [43]. GABA is a potential signaling molecule and metabolite involved in various physiological and biochemical reactions such as intracellular pH osmoregulation, the tricarboxylic acid cycle, and carbon and nitrogen balance [18]. High levels of GABA have been found in several plants to improve tolerance to different abiotic and biotic stresses [44,45]. GABA enters the TCA cycle through the conversion of the γ-aminobutyric acid transporter (GABA-T) as well as succinate dehydrogenase (SDH), a key step in GABA metabolism. Our results showed that GDH enzyme activity was significantly enhanced in the leaves and roots of tall fescue under Cd treatment, which helped dehydrogenate α-KGDH in the TCA cycle to synthesize Glu, resulting in a large intracellular enrichment of Glu. The Glu content was similarly increased in *Ageratum conyzoides* and *Crassocephalums crepidioides* under higher concentrations of Cd stress in the study by Zhu et al. and the accumulation of Glu may be the result of coordination with other amino acids in response to the stress [46]. Cd-stressed rice supplemented with Glu (3 mM) significantly reduced Cd accumulation in roots and stems, alleviated Cd-induced oxidative stress, reduced H_2_O_2_ and MDA contents, and increased the activity of antioxidant enzymes. The main energy metabolic pathway, the TCA cycle, was inhibited under CdCl_2_ stress, and the GABA branch was activated to regulate the plant carbon–nitrogen balance [47]. The GABA content of both the leaves and root parts of tall fescue decreased significantly under Cd stress, especially the leaf GABA content, which decreased by 62.15%, while the SDH enzyme activity increased significantly. The leaf GABA shunt also showed a higher contribution in the MFA, while the root GABA shunt was lower than the other groups (Figure 7B). The strong increase in GABA in the plant response to cadmium stress may lead to a Glu deficiency, resulting in an insufficient supply of basic processes such as chlorophyll and metabolite synthesis. The addition of GABA reduces the energy consumption of endogenous GABA synthesis in response to stress, provides a carbon source, and may act as a stress-resistant signal to regulate plant metabolism. The enhancement of SDH enzyme activity accelerates the utilization of GABA to succinic acid in the TCA pathway to maintain a metabolic balance in the body.

## 4. Materials and Methods

### 4.1. Plant Materials and Treatments

The test material was the tall fescue variety ‘Ruby II’, purchased from the Beijing Crawford Grass Company (Beijing, China). The purity and germination rates of the seeds were above 95%. The conditions for the plant culture incubator were set as follows: the temperature during the day/night was 25 °C/20 °C, the relative humidity was 60%, and the light (700 μmol m^−2^·s^−1^ PAR)/dark time was 14 h/10 h. After 10 days of germination, seedlings of uniform growth were selected and transferred to shade containers filled with 1/4 Hoagland nutrient solution for hydroponic cultivation, with 50 holes per box and 30 plants per hole. The nutrient solution was changed every 3 days (the pH was maintained between 5.6 and 6.5). Different treatments were used for plants after two days of hydroponics. The treatments in the experiment were a control (C), γ-aminobutyric acid (GABA 0.1, 0.5, 1, 5 and 10 mmol/L), Cd 20 (20 μmol/L), Cd 20 + GABA (0.1, 0.5, 1, 5 and 10 mmol/L), Cd 50 (50 μmol/L), and Cd50 + GABA (0.1, 0.5, 1, 5 and 10 mmol/L), with three replicates for each treatment. The above scheme was used further to determine the concentration of external GABA and Cd stress. The C group was treated with distilled water. The GABA treatment was prepared by dissolving GABA powder in water to make a concentrated solution. The treatment solution was changed every 3 days and samples were taken on day 11 to determine the GABA and Cd stress application concentration. The new material was treated after 2 days of acclimatization to hydroponic growth. Four treatments were tested, C (control), GABA (0.5 mmol/L GABA), C + Cd (20 Cd), and GABA + Cd (0.5 mmol/L GABA + 20 μmol/L Cd), with three replicates of each treatment. Samples were taken on days 3, 7, 11, and 15 of treatment to find the effect of exogenous GABA on Cd stress of tall fescue, snap-frozen in liquid nitrogen, and stored in an ultralow temperature refrigerator at −80 °C for backup.

### 4.2. Measurement of Root and Leaf Length

For each treatment, ten *Festuca arundinacea* plants were randomly selected, meticulously cleaned with distilled water, and gently dried to remove surface moisture. The plants were then carefully arranged flat on a root-sweeping tray, and a photograph was captured directly above each plant. Subsequently, the Digimizer software V800 (Epson Perfection V800 Photo) was employed to measure the plant height and primary root length from the captured images.

### 4.3. Measurement of Cd Content

Cd content was determined by the method of Song et al. [48] with slight modification. We weighed 0.2 g of dried, ground, and sieved roots/leaves in a digestion tube for microwave digestion. The program was 80 °C for 1.5 h, 120 °C for 2 h, and then increased to 160 °C for 1.5 h. Then, the digestion solution was fixed in 25 mL to be measured. The Cd content was then measured using an inductively coupled plasma mass spectrometer (ICP-MS, Thermo Fisher RQ, Waltham, MA, USA).

### 4.4. Measurement of Photosynthetic Pigments

Photosynthetic pigments were determined using the method of Lichtenthaler [49] with slight modification. Fresh leaves (0.05 g) were taken, washed in distilled water and dried on the surface, cut up, and placed in a 10 mL centrifuge tube. Then, 8 mL of 95% ethanol was added, and the leaves were completely immersed in the ethanol solution and left to stand for 48 h protected from light. The absorbance values of the extracts at 665 nm, 649 nm, and 470 nm were measured. The absorbance values were then used to calculate total chlorophyll, chlorophyll a, chlorophyll b, and carotenoid contents.

chlorophyll a = 13.95A665 − 6.88A649 (mg/L)

chlorophyll a = 13.95A665 − 6.88A649 (mg/L)

chlorophyll b = 24.96A649 − 7.32A665 (mg/L)

total chlorophyll = chlorophyll a + chlorophyll b (mg/L)

carotenoid = (1000A470 − 2.05chlorophyll a − 114.8chlorophyll b)/245 (mg/L)

### 4.5. Measurements of MDA Content and Antioxidant Enzyme Activities

Ten tall fescue plants were randomly selected, rinsed in distilled water, and dried. They were then photographed, with the plants lying flat on a root-sweeping tray directly above them, and the plant height and primary root length were measured using Digimizer software. The malondialdehyde (MDA) content was determined by the thiobarbituric acid colorimetric method [50]. The activities of catalase (CAT), peroxidase (POD), and ascorbate peroxidase (APX) were determined using enzymatic kinetic methods. Fresh leaves or roots (0.3 g) were frozen and ground to a powder; 2 mL of enzyme extract (pH 7.0) was added and ground thoroughly to form a slurry and transferred to a 10 mL centrifuge tube; the supernatant was centrifuged at 15,000× *g* for 20 min at 4 °C, transferred to a 4 mL centrifuge tube, and stored at 4 °C. The supernatant was transferred to 4 mL centrifuge tubes and stored in a refrigerator at 4 °C. The absorbance change at 240 nm was measured for CAT enzyme activity, at 470 nm for POD enzyme activity, and at 290 nm for APX enzyme activity [51,52].

### 4.6. Measurements of the Major Metabolites of the GABA Shunt, and Related Enzymes Activities

The micro-method was employed to determine the contents of γ-aminobutyric acid (GABA) and glutamic acid (Glu), and the activities of glutamate dehydrogenase (GDH, EC 1.4.1.2) and succinate dehydrogenase (SDH, EC 1.3.5.1). The specific measurement procedures were conducted in accordance with the instructions provided in the corresponding reagent kits (Kemin Biotech, Suzhou, China).

## 5. Statistical Analysis

The SPSS 23 (IBM, Armonk, NY, USA) was used to analyze the data. Significant differences among treatments were tested by using a one-way analysis of variance for the least significant difference (LSD) between significant differences at *p* < 0.05. A multiple factorial analysis (MFA) was performed with the R package FactoMineR (version 2.3) [42,53].

## 6. Conclusions

In summary, the results of this study indicate that the external application of GABA (0.5 nmol/L) can alleviate the growth impairment of tall fescue caused by Cd stress. The results showed that GABA pretreatment significantly increased photosynthetic pigment content after 15 d of stress to promote photosynthesis, thereby enhancing antioxidant capacity by regulating the activities of antioxidant enzymes (APX, CAT, POD) that contribute to the scavenging of ROS, thus effectively alleviating oxidative stress damage under Cd stress conditions. In addition, the GABA shunt was activated in GABA-treated tall fescue, and the GABA content was increased as a nutrient in response to Cd stress. GABA has great potential as an exogenous additive for regulating plant growth and stress tolerance, and future research could focus on the application of GABA in plants to cope with complex stress environments and its coupling with management measures such as fertilization.

## Data Availability

The original contributions presented in this study are included in the article material. Further inquiries can be directed to the corresponding author(s).
